# Motor performance in a shape sorter task: A longitudinal study from 14 to 36 months of age in children with an older sibling ASD

**DOI:** 10.1371/journal.pone.0217416

**Published:** 2019-05-28

**Authors:** Fabrizio Taffoni, Valentina Focaroli, Flavio Keller, Jana Marie Iverson

**Affiliations:** 1 Laboratory of Biomedical Robotics and Biomicrosystems, Università Campus Biomedico di Roma, Rome, Italy; 2 Laboratory of Developmental Neuroscience, Università Campus Biomedico di Roma, Rome, Italy; 3 Infant Communication Lab, Department of Psychology, University of Pittsburgh, Pittsburgh PA, United States of America; Istituto di Fisiologia Clinica Consiglio Nazionale delle Ricerche, ITALY

## Abstract

During development, motor skills are fundamental in supporting interactions with the external world. The ability to plan actions is a particularly important aspect of motor skill since it is involved in many daily activities. In this work, we studied the development of motor planning longitudinally in children with an older sibling with Autism Spectrum Disorder (ASD) who are at heightened risk (HR) for the disorder and children with no such risk (low risk; LR) using a shape sorter task. Children were observed at 14, 18, 24 and 36 months. Three HR children with a later diagnosis of ASD (HR-ASD) were analyzed separately from the rest of the sample. Behavioral and kinematic data indicated that precision demands significantly influenced children’s actions, and that children’s performance improved with age. No differences were found between the HR and LR groups, but a descriptive analysis of data from the three HR-ASD suggested differences in the variables describing children’s action (as reaching time and acceleration) as well as variables describing children’s performance (as the adjustment of the shapes).

## Introduction

The ability to interact with the environment and to explore the world around us is a key ingredient in early development: not only does it promote the acquisition of new motor skills, but it also supports development in other fundamental domains (e.g., cognition, communication; e.g., [1]). The progressive acquisition of new motor skills provides infants with enriched possibilities for interacting with the environment and promotes the acquisition of language and the development of social skills [2, 3].

These motor capabilities depend both on the development of the body and on the acquisition of appropriate motor control strategies. Reaching is one of the first actions performed by infants. Skilled reaching and object manipulation require postural development and improvements in the control of the head and trunk. This process starts with the ability to maintain head position at midline [4], which stabilizes the visual field and allows infants to focus on targets toward which reaching will eventually be directed. Subsequently, refinements in postural stability support more precise reaching performance [5].

Motor planning plays a fundamental role in the refinement of reaching. One widely described phenomenon of motor planning is that in an action sequence, the final goal influences the way in which individuals perform the initial action, suggesting that the initial action is planned according to the complexity of the final goal. Several studies of adults have shown how characteristics of the final goal of the action influences motor planning. Pioneering research by Fitts [6], showed that the time to reach a target depends on the distance from the object and on its size: the larger and closer the object, the faster is the act of reaching. Marteniuk et al. [7] studied the kinematics of reaching in two different conditions: adults were asked to grasp an object and place it in a large tub (simple condition) or in a tight fitting well (precise condition). Their results confirmed Fitts’ observations: reaches were slower in the precise compared to the simple condition. Similarly, some studies (e.g. [8, 9, 10, 11]) hypothesized that if the grip movement involves a subsequent goal action, the duration of the initial reaching movement will be significantly less than a movement that does not anticipate a goal. What emerges is a planning and a pre-motor evaluation that begin well before the act of grasping and evident during the reach to the target. This phenomenon is also called “second order planning” and suggests that actions are organized around intentions, in particular, on what the person intends to do with the object in the immediate future [12, 13].

The development of reaching in infancy is an important milestone and becomes efficient over an extended period of time. In particular, changes in infants’ reaching have been described as a two-phase process. Between 3 and 5 months of age, the first phase begins. This phase primarily involves motor exploration, which allows infants to learn how to adjust the speed of the movement to the task at hand [14]. From 6 months of age, many improvements appear. The infant starts to grasp objects taking into account their physical properties as well as integrating movement planning. This implies that 6-month-old infants are able, for instance, to change the kinematics of their reaching whether they reach to grasp versus to simply touch the same toy [15]. In addition, in this phase, infants learn how to: (i) match the orientation of their hand with the orientation and shape of the target object during the transport phase of the reach [16, 17]; and (ii) use one or two hands to adapt their reach and grasp to the sizes of objects [18, 19, 20]. Then, by the age of 10 months, they start anticipating two concurrent dimensions of objects, such as orientation and size, although their adjustments may be sequential [21, 22].

Claxton and colleagues [23] studied motor planning in infants and compared 10-month-old infants’ reaches for a ball when it was placed into a tub (imprecise condition) vs. into a narrow tube (precise condition). Reaching for the ball was slower when infants had to put the ball in the tube compared to trials in which they placed it in the tub. Chen et al. [24] extended these results to toddlers aged 18 and 21 months. In their study, children interacted with a set of blocks that were either to be placed into a large tub or used to build a tower. They also found differences in the kinematic variables measured during the reach phase: when the cubes were used to build a tower, the reaching to grasp movement was slower than that measured when cubes were simply put into the tub. Taken together, these findings indicate that more sophisticated reaching skills emerge by the end of the first year and continue to develop through the second year of life.

The experimental tasks described above examined motor planning skill via goal directed actions in which children interacted with the same object, placing it on a target location and with two levels of precision demand. Örnkloo and von Hofsten [25] took a somewhat different approach, studying the development of children’s motor planning ability in a task requiring the insertion of objects into apertures. All objects were the same length but had different cross sections; thus, the task had more than two levels of difficulty. Task demands varied according to the different cross sections of the objects (circular, square, rectangular, elliptical, triangular) and on the orientation in which they were presented. Objects were presented in a simpler vertical orientation (insertion of the object into the hole involved only a translation) on some trials, and in others they were presented in a more difficult horizontal orientation (involving translation and rotation of the block). Effective execution of the task required children to perceive the spatial relationship between the object and the aperture and to rotate the object appropriately in anticipation of the final goal. Eighteen-month-old children understood that the task required insertion of the object into the opening, but they found it difficult to accomplish, especially when the block was presented horizontally. They moved the object without rotating it over the hole and tried to insert it. By 26 months, toddlers were able to plan the movement and to rotate the object before reaching the aperture on the box.

Ornkloo & von Hofsten [25] mainly focused their study on the analysis of motor performance during placement movement (i.e. when children have grasped the object to move it toward the box) and using a cross-sectional design. What remains unclear is whether children modify the way in which they reach for the objects in relation to the level of difficulty of the final target, and how this skill develops with age. In the present study, we address this issue by utilizing a longitudinal design and studying both the reaching and placement phases of children’s actions.

We also included a group of children known to be at risk for early delays and disruptions in fine motor skills to explore whether their performance in this task differed from that of peers with no such risk. Infants who have an older sibling with autism spectrum disorder (ASD) are at heightened biological risk (HR) for developing the disorder and for other developmental delays than are children with a typically-developing older sibling and no family history of ASD (Low Risk; LR; [26, 27]). HR children show high inter-individual variability in multiple developmental domains [28, 29]; and there is also ample work indicating the presence of delayed fine motor abilities among HR infants (e.g., [30, 31, 32]). In addition, there is some evidence of differences in motor planning among HR children. Focaroli et al. [33] used the ball in the tube and tower building tasks employed by Claxton et al. [23] and Chen et al. [24] in a longitudinal study of HR and LR children from 18 to 36 months. They also placed magneto inertial sensors on children’s wrists to gather kinematic data on reaching. Findings indicated that HR children reached for the cubes more slowly (i.e., mean acceleration was lower) than LR children, but only in the tower building task.

In light of these results, we wished to extend this research by examining motor planning performance in HR compared to LR children in a task that required a different goal action (insertion of an object) and in which task demands varied along a continuum of difficulty. In the present longitudinal study, we administered the task described by Örnkloo and von Hofsten [25] to HR and LR children at 14, 18, 24, and 36 months. We placed magneto-inertial sensors on their wrists and inside the shape blocks they manipulated in order to gather data on the kinematics of reaching and the orientation of the block during insertion attempts, variables not previously examined by Örnkloo and von Hofsten [25]. While this task does not involve comparisons between two conditions that differ in precision demands (as in [23] and [24]), it provides an opportunity to examine the coordination of the action sequences necessary to accomplish it [34]. The different cross sections of the blocks and the two presentation orientations allowed us to define a progression along a continuum of difficulty. Incorporation of the sensors permitted collection of quantitative information on the action sequences, and the longitudinal design allowed us to describe the development of planning skills necessary to accomplish the task effectively.

## Material and methods

### Participants

This research was approved by the University of Pittsburgh Institutional Review Board. We enrolled 33 children in the study: 19 (10 males) were HR children and 14 (9 males) were LR children. All children in both groups were born full-term from uncomplicated pregnancies and deliveries and came from English-speaking homes.

The HR children were part of a larger longitudinal study on the early development of HR infants (e.g., [35, 36]). Their families were recruited through a university-based Autism Research Program, parent support organizations, and local agencies and schools serving families of children with ASD. Prior to infant enrollment in the larger study, the Autism Diagnostic Observation Schedule (ADOS; [37]) was administered to all older siblings by a trained clinician to confirm their diagnosis. At 36 months, HR children were seen for final diagnostic assessment and classification by an experienced clinician blind to all previous study data using the ADOS and DSM-IV criteria. Three HR children (2 males) received an ASD diagnosis. For this reason, their data were considered separately and were not included in the HR sample for group analyses. An additional HR participant was excluded since s/he refused to complete the task. Therefore, the final HR sample included 15 children (9 males).

The comparison group of 14 LR children was recruited via advertisements in local parent magazines, newsletters, neighborhood circulars, pediatricians’ offices, daycare and preschool centers, neighborhood email distribution lists, and word of mouth. All LR children had a typically developing older sibling and no family history of ASD (i.e., no first or second degree relatives diagnosed with ASD).

### Procedure

Prior to the start of the study, parents of the children signed a written consent form describing the purpose of the study. Children were visited at home at 14, 18, 24, and 36 months of age: LR children were seen on or within a few days of the monthly anniversary of their birthday, while HR children were seen at a time different from regularly scheduled visits for the larger study (for further description of the procedures employed in the larger study, see [36]).

**Fig 1:**
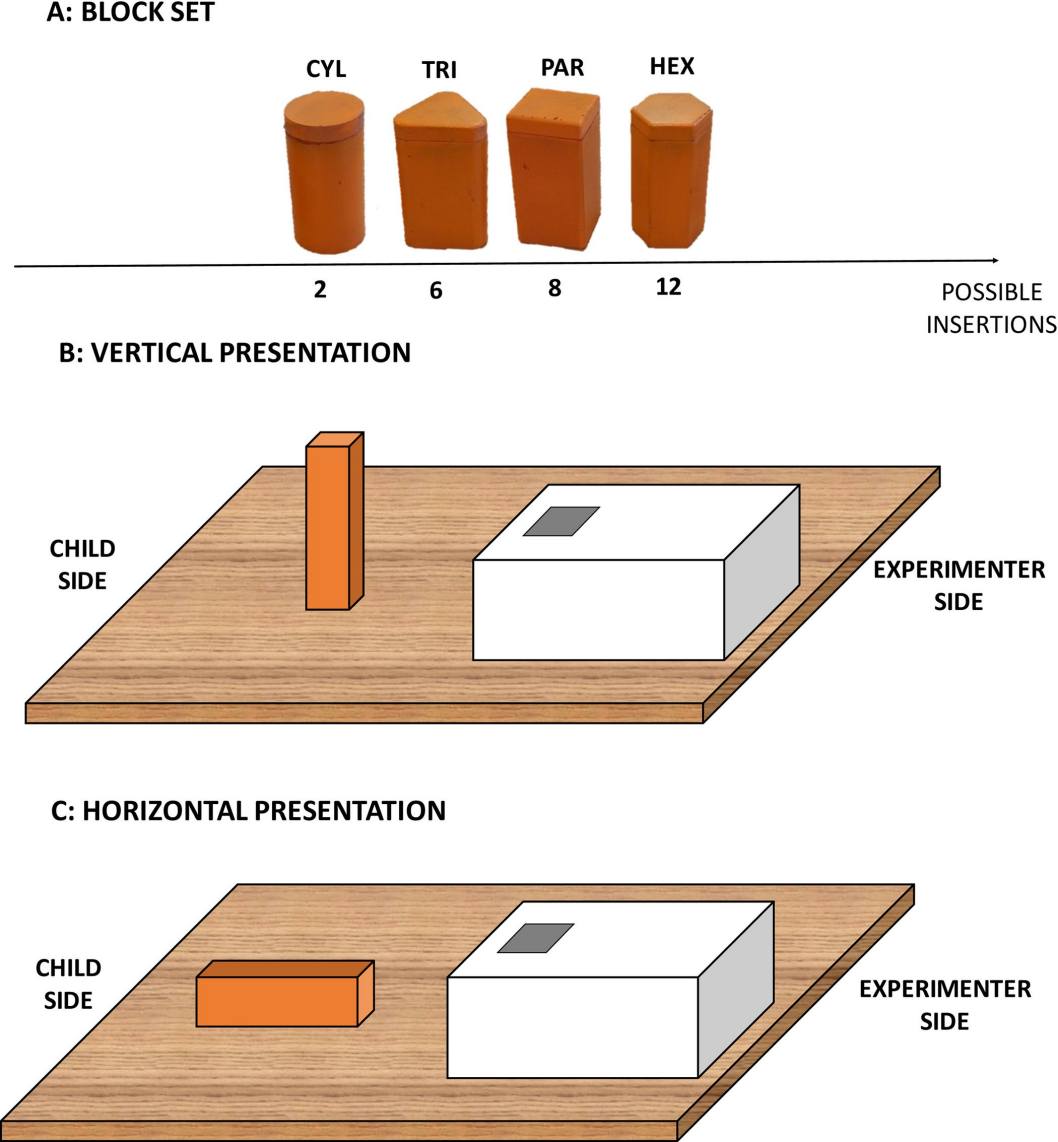
The shape sorter protocol. A) Blocks used in the protocol. The cross section of each block defines the possibilities of insertion into the aperture: the cylinder may be inserted in two ways, from the upper base or from the lower base; the triangular prism in six ways, three for each base; the parallelepiped in 8 ways, 4 for each base; and the hexagonal prism in 12 ways, six for each base. B) and C) show examples of vertical and horizontal orientation of the parallelepiped block respectively.

Study observations were conducted in families’ homes. A primary experimenter interacted with the child in a quiet room in the presence of a primary caregiver (usually the mother), while a second experimenter video recorded the session. Children sat on the floor or on their mother’s lap in front of the primary experimenter, who administered the task. Children were asked to insert a block into a box placed centrally in front of them and within reaching distance, which varied slightly across children and sessions. Because we observed children over a 22-month period, their body dimensions changed substantially during the course of the study, and thus we chose not to use a fixed distance between the hand and the block. All blocks were of the same height (90 mm). The box was covered with a lid containing an aperture corresponding to the shape of the presented block. The aperture was 1 mm larger than the base of the block. We used four different blocks: a cylinder (CYL, circular base, radius r = 23mm), a parallelepiped (PAR, square base, side l = 45mm), a triangular prism (TRI, triangular base, side l = 50mm), and a hexagonal prism (HEX, hexagonal base, side l = 25mm). Blocks therefore varied in the number of possibilities for insertion, with more complex shapes (e.g., HEX) requiring greater precision (see Fig 1A).

Each block was presented in two different orientations. In vertical presentation (VER, Fig 1B), which was administered first, the block was placed on its base, aligned with the aperture. In horizontal presentation (HOR, Fig 1C), presented second, the block was placed on one of its lateral faces (in the case of the cylinder, the block was placed on a small support to prevent it from rolling away). Prior to administering the task, the experimenter showed children how to insert each block. Subsequently, children were asked to replicate the task: if children refused to perform the trial or failed on two consecutive attempts, the experimenter moved to the next trial.

Fig 1 reports the sequence of shapes presentation. Participant received a total of 24 trials (3 trials per orientation, with 2 different orientations, per each of the 4 shapes).

**Fig 2:**
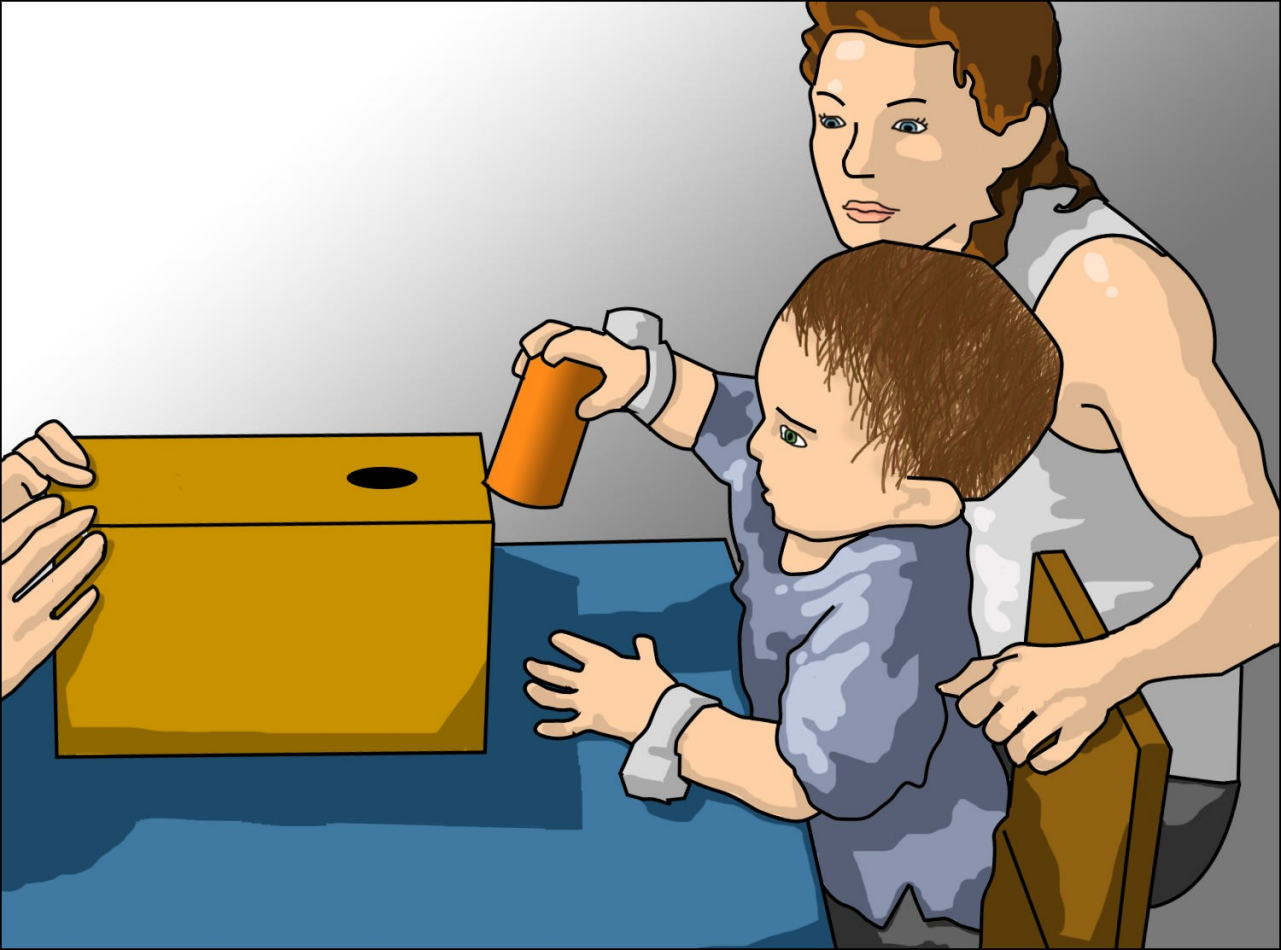
Experimental scenario.

Blocks were instrumented with a 9-axis magneto-inertial device to reconstruct their orientation [38]. The block sensor contains a Bluetooth class II module (Parani-ESD200, Sena Technologies) to allow wireless real-time communication. Children wore two wrist bracelets instrumented with two additional magneto-inertial devices to measure reaching kinematics [39]. The bracelets were cabled and connected to a remote PC where a custom Labview program managed data acquisition and synchronization. All experimental sessions were video recorded by a camera (Canon Vixia HF M52) recording at a rate of 29.97 frames/sec. Fig 2 shows the scenario of the experimental setting.

### Coding and variable creation

For each trial, behavioral data were extracted from videos and kinematic data from the wrist and block sensors.

Videos were coded by a team of coders naïve to children’s risk status (HR or LR) using ELAN Version 5.0.0-beta [40] software [41]. Prior to commencing independent coding, all coders were trained to a criterion of 80% agreement on three consecutive training videos. On each trial, coders focused two specific motor acts: reaching and placement. As in previous work [33], *reaching* began at the first frame in which the child moved the hand from the surface and ended at the first frame in which the hand made contact with the block. *Placement* began in the first frame in which the child lifted the block from the table and ended when the child released it into the box. For both reaching and placement phases, coders extracted the respective durations. They also noted when the child touched the box lid with the block for the first time. We used this time to estimate the orientation of the block with respect to the aperture, as in [25].

Kinematic data were low pass filtered at 20 Hz. Filtered data from the wrist bracelets were used to calculate mean accelerations during reaching and during placement. Data from the block sensor were used to calculate *vertical error* (i.e. how much the longitudinal axis of the block object was tilted with respect to gravity) and *horizontal error* (i.e. the misalignment between the cross-section of the object and the aperture on the lid; for additional details, see [42]).

### Statistical analysis

Not all participants contributed the same numbers of trials, since we necessarily excluded trials in which children changed hands between reaching and placement and trials in which children performed a reach but did not insert the shape into the box. The percentages of invalid trials at each age were as follows for the LR and HR groups respectively: 17% vs. 20% at 14 months, 9% vs. 15% at 18 months, 2% vs. 18% at 24 months, and 4% for both groups at 36 months. For this reason, we utilized random effects regressions for our data analyses. This technique allows consideration of multiple data points from the same participant as separate measures and avoids averaging repeated measures for each participant [43]. It also accounts for interdependency and structuring of the data and is particularly suited for analyzing behavioral and ecological data that typically have one or more levels of aggregations.

The dependent variables were reaching duration, placement duration, mean acceleration of reaching, vertical error and horizontal error. Random effects regression models were computed separately for each dependent variable, and with group (HR, LR), age (14, 18, 24, 36 months), shape (CYL, PAR, TRI, HEX), and orientation (VER, HOR) as predictors and participants as a random factor.

## Results

In this research, we examined the development of motor planning in an object fitting task. Our aim was to study planning skill during reaching and object placement and its development longitudinally in HR and LR children. Statistical analyses compared performance and patterns of age-related change between the HR and LR groups between 14 and 36 months of age.

Because only 3 participants received an ASD diagnosis, it was not possible to perform reliable inferential statistics to compare their performance with that of the HR and LR groups. Thus, we followed prior research designed to identify features of ASD and ASD risk with similar sample sizes [44, 45] and present mean plots for these infants.

Initial analyses revealed no significant differences between the LR and HR groups on any of the dependent variables. For this reason, we focused on the overall sample of 29 children without regard to risk status and investigated potential effects of the other independent variables considered here (Age, Shape, Presentation). Results for these three variables are presented in turn below. All statistical analysis were run on HR and LR groups only. Table 1 reports descriptive statistics for all dependent variables at all ages for the LR and HR groups.

**Table 1:** Mean and standard deviations for all variables at all ages.

		14 months		18 months		24 months		36 months	
		M	SD	M	SD	M	SD	M	SD
Reach Duration [s]	LR	0.8	0.63	0.74	0.18	0.56	0.22	0.51	0.16
	HR-ND	0.77	0.39	0.86	0.74	0.64	0.22	0.48	0.18
Place Duration [s]	LR	5.98	4.81	6.98	55.96	5.01	3.61	3.63	2.5
	HR-ND	6.61	5.45	5.77	4.04	4.57	3.17	3.3	2.35
Mean Reach acceleration [m/s^2]	LR	2.85	1.52	3.15	1.84	2.45	2.11	2.84	1.47
	HR-ND	2.76	2.71	3.02	1.66	2.62	0.83	2.53	1.6
Horizontal Error [°]	LR	15	10.13	16.9	11.45	16.3	7.58	11.5	7.38
	HR-ND	15.21	14.47	18.14	9.65	19.46	11.8	13.81	8.82
Vertical Error [°]	LR	44.33	24.62	48.26	16.93	47.48	19.86	53.87	17.01
	HR-ND	51.78	25.22	48.84	16.48	46.53	18.15	49.38	20.54

### Age effects

We began by examining the effect of age on our measures of reaching, placement, and block orientation. Based on prior cross-sectional work [25], we expected to find improvements in children’s performance with age.

**Fig 3:**
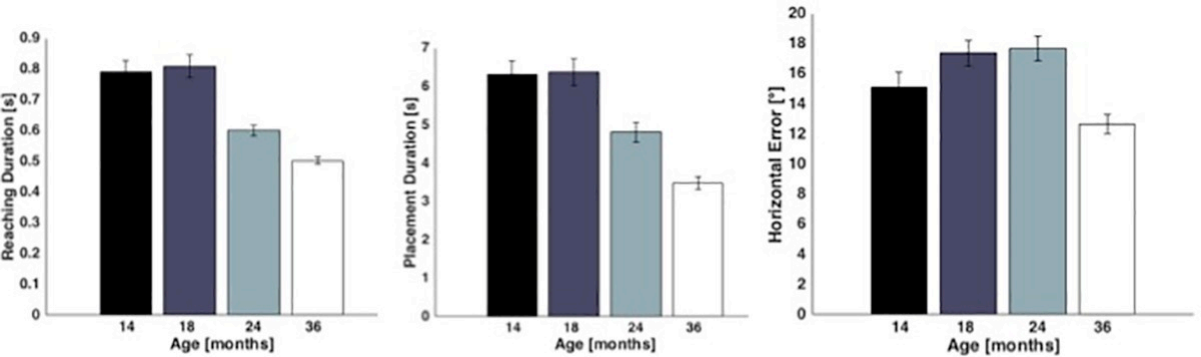
Significant age effects: Means±SE are reported.

Consistent with our prediction, analyses revealed an effect of age on reaching (z = -8.64 p<0.01, Fig 3A) and placement duration (z = -8.84 p<0.01, Fig 3B). With increasing age, children took less time to perform both reaching and placement actions. No significant effects of age on mean reaching acceleration were found. Finally, there was a small but significant increase in horizontal error with age (z = 2.26 p<0.02, Fig 3C) from 14 to 24 months of age, with a sharp decline by 36 months. This result suggests that the ability to control object orientation is gradually refined during the third year of life.

### Shape effects

As noted above, variation in block shape may affect task performance because the effort required to fit the block into the aperture depends on the block’s cross-section. The CYL has no preferential horizontal orientation; it only requires alignment of the block vertically with respect to the opening. For this reason, horizontal error cannot be defined for this block because the child has only 2 possibilities for insertion (i.e., inserting one of the two bases). The PAR, TRI prism, and the HEX prism have 8, 6, and 12 possibilities for insertion respectively.

**Fig 4:**
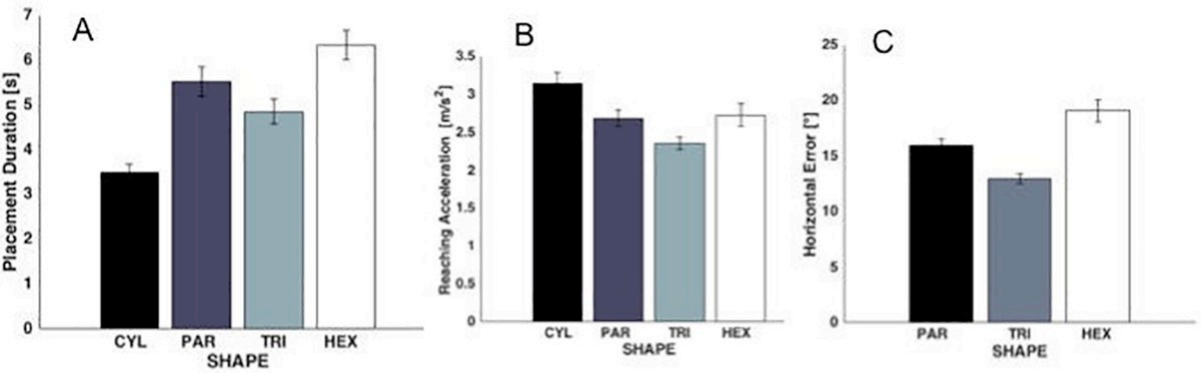
Significant shape effects: Means±SE are reported. Shapes are reported in the presentation order. The horizontal error (C), is cannot be defined for CYL.

Analyses indicated that the number of possible insertions had a significant effect on children’s planning during reaching and placement execution. There was also an effect of shape on reaching acceleration (z = -2.49 p<0.01, Fig 4B), which decreased with increasing numbers of possible insertions, indicating that children’s reaching movements were slower when difficulty was greater. Placement duration increased as number of possible insertions increased (z = 5.36 p<0.01, Fig 4A). As difficulty of insertion increased, children required more time to transport the shape to the box and successfully insert it into the opening. Finally, horizontal error (z = 1.86 p<0.06, Fig 4C) increased with the number of possible insertions, indicating a greater tendency to misalign the cross section of the block with the aperture in the lid.

### Orientation effects

**Fig 5:**
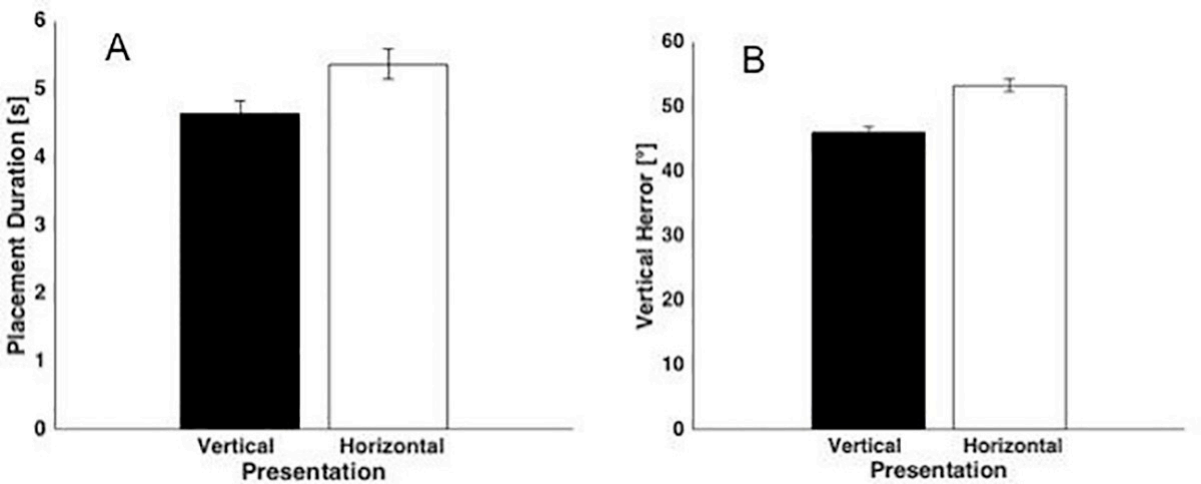
Significant orientation effects: Means±SE are reported: A) effect of orientation on placement duration; B) effect of orientation on Horizontal Error.

The initial orientation in which the block was presented may influence planning demands for reaching and placement actions. In particular, in the vertical presentation, the block is oriented with its longitudinal axis vertical and with the base horizontally aligned with the aperture, so children only need to transport the block to the aperture and release it. Horizontal presentation requires greater planning effort since children must perform two rotations. They must first align the longitudinal axis of the block with gravity, and then they have to adjust its horizontal orientation, aligning the cross-section of the block with the box aperture. As expected, placement duration (z = 3.75 p<0.01, Fig 5A) and vertical error (z = 3.42 p<0.01, Fig 5B) were significantly higher in the horizontal relative to the vertical orientation. In other words, when children had to insert a block presented horizontally (i.e. in the orientation requiring more planning effort) into the box, they required more time and their capacity to control the vertical alignment was poorer with respect to performance with vertically-oriented objects.

### HR children diagnosed with ASD

**Fig 6:**
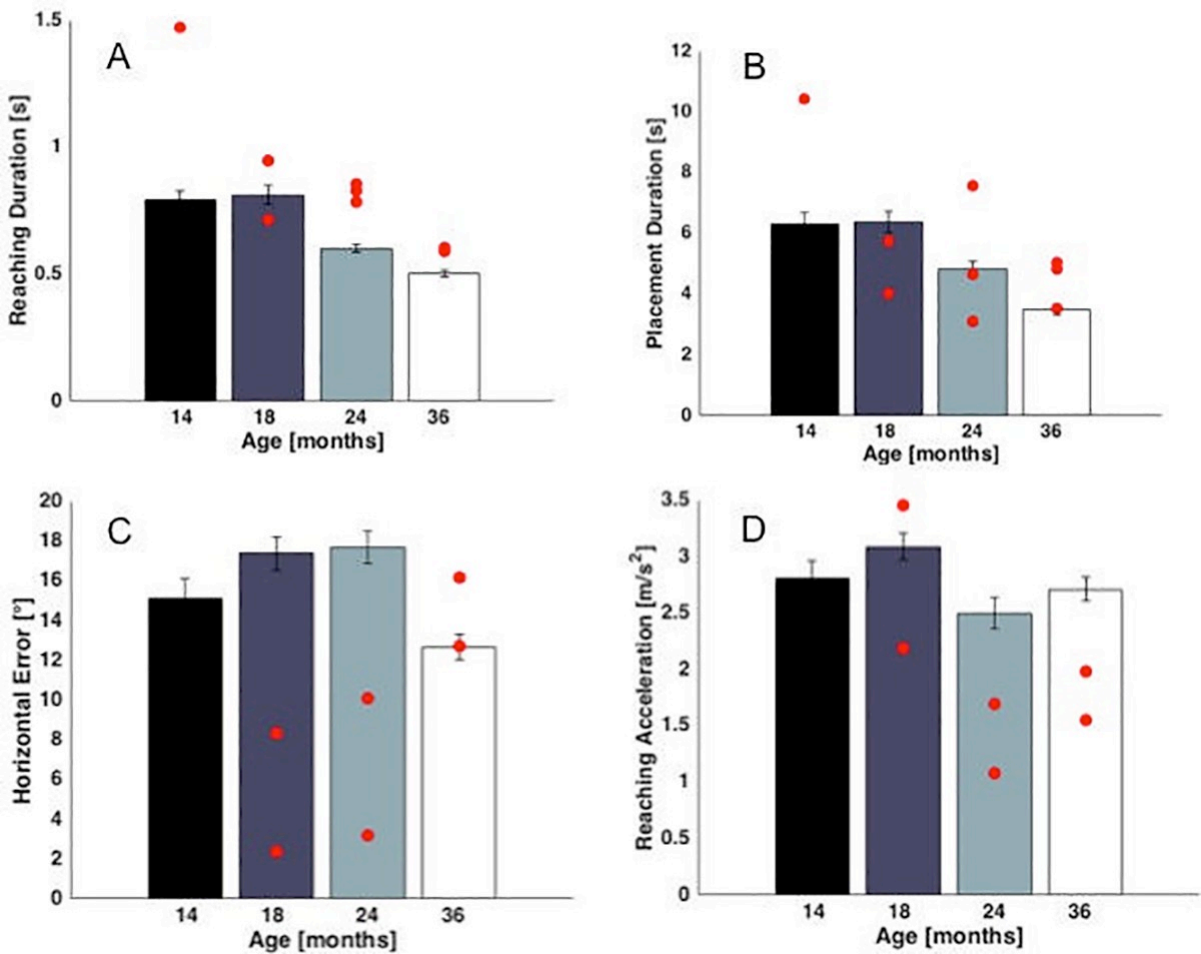
Age effects on LR+HR children (columns represent means and error bars the standard error) and the 3 HR-ASD children (red circles). Figures A-C report the observational data for which age effects were observed. One HR-ASD child was tested at 14 months and 2 were tested at 18 months. Figure D reports the kinematics of reaching: 2 HR-ASD children wore the sensors. No sensor data were available for the 14-month observation.

As noted above, due to their small number, data from the three HR children diagnosed with ASD (HR-ASD) were examined separately. We calculated descriptive statistics for all variables for which we found age effects in the combined HR/LR group (i.e. reaching duration, placement duration and the horizontal error) displayed in Fig 3 for each HR child diagnosed with ASD. Below we present the individual data for each HR-ASD child, represented as red circles in the figures (see Fig 6A and 6B). We also present kinematic data on reaching acceleration for 2 of the 3 children (one child consistently refused to wear the wrist sensors; see Fig 6C and 6D).

As is evident in the figures, the three HR-ASD children seem to require more time when reaching for objects: 8 of the 9 measures were greater than the mean reaching times of the overall group at all ages (Fig 6A). Placement duration was more variable and no consistent pattern was apparent (Fig 6B). Fig 6C and 6D presents the variables calculated from the wrist sensors. Horizontal error was lower than the average values for the overall group until 24 months of age, but this was reversed by 36 months (Fig 6C). Reaching acceleration values were also generally lower, consistent with the higher durations of reaching obtained for the HR-ASD children (Fig 6D).

## Discussion

In this research, we studied the development of the ability to plan actions involving the transport, alignment, and placement of objects longitudinally in HR children with familial risk for ASD and LR children with no such risk using the shape sorter task devised by [25]. Performance in this task reflects a constellation of developing motor, perceptual, and cognitive skills. An innovative feature of this study was the use of magneto-inertial sensors worn by the child and housed in the shape blocks. This approach allowed us to collect quantitative data not only on the kinematics of reaching and placement, but also on the orientation of the block. In addition, while previous research has focused primarily on the placement phase of children’s actions, we also examined the reaching phase to assess planning skills prior to object insertion.

There were no significant group differences between HR and LR children on any of the variables examined here. This was surprising in light of previous findings of slower reaching movements in HR compared to LR children in a tower building task [33] and reports of delays in fine motor skills in HR infants (e.g., [32]) and persistent motor difficulties in school aged HR children (e.g., [3]). Moreover, studies of adults with ASD have reported difficulties with gross and fine motor skills (e.g. [46, 47]). Glazebrook and colleagues [48] found that young adults with ASD spent more time preparing and executing manual movements than a comparison group in a fine motor task. They showed slower movement times in reaching in kinematic analyses.

The absence of group differences in our study might be explained by the nature of the task itself. The tower building task, in which group differences were apparent, involves two different goal actions (throwing the cube in a tub vs. placing it on top of another cube) that vary in level of difficulty. In the shape sorter task, however, children were always asked to perform the same action with the same goal (i.e., to precisely insert a block into a corresponding aperture of the same shape). Because the shapes varied in number of possible insertions and the starting orientations varied in number of translations required prior to insertion, level of difficulty varied along a continuum, and differences from one shape to the next are less distinct.

We observed a general improvement in action efficiency with age, with both reaching and placement durations decreased significantly across observations. However, we did not find a corresponding improvement in spatial planning. Rather, horizontal error increased up to the 24-month-visit and then dropped to its lowest value at 36 months. This finding is consistent with the observations of Ornkloo and von Hofsten [25], who reported improvements in the control of horizontal orientation at 24 months of age. This ability is subsequently consolidated, which could account for the reduction in the horizontal error at 36 months of age in our data. In addition, we observed an increase in placement duration in relation to object shape and initial orientation. Placement duration increased with the number of possible insertions of the block, and children needed more time to place the block into the aperture when it was presented in the horizontal orientation. Similar differences were observed for horizontal and vertical error. Horizontal error depended on the cross section of the block: the higher the number of possible insertions, the higher the error value. Moreover, vertical error was greater when blocks were presented horizontally. And consistent with previous findings (i.e. [23, 24]), the kinematics of the reaching phase were impacted by increases in task difficulty: wrist acceleration values decreased as number of possible insertions increased, suggesting that planning demands were greater on these trials. Schmidt and colleagues [49] proposed a model for rapid motor tasks (lasting less than 200 ms) and well-learned tasks (i.e. which do not require feedback) lasting up to 500 ms. This model predicts the accuracy of the movement (i.e. motor output variability) from the impulse for acceleration: the lower the accuracy, the higher the amplitude of the impulse for acceleration. They also suggest a possible interpretation of motor output variability for longer motor tasks. In these cases, they proposed a very large initial acceleration impulse, the monitoring of the errors that this variability has produced, and a correction of the errors to increase motor accuracy. This interpretation accounts for both the reaching acceleration amplitude, which decreases as task accuracy increases, as well as the variability of reaching amplitude, which decreases with age, i.e. with the development and tuning of sensory feedback system and control.

Finally, comparison of the performance of the combined HR/LR group to that of the three HR-ASD children revealed that they tended to take more time when reaching for objects and to execute reaching movements with a lower acceleration. Moreover, until 24 months of age, horizontal error for the HR-ASD group was considerably lower than the average value of the overall group, but this tendency reversed by 36 months, the age at which LR children appear to master the ability to pre-adjust the shape orientation to fit with the aperture [25]. Although these data come from a very small number of children and must therefore be interpreted with great caution, they are nevertheless consistent with other reports that performance of children with ASD differs from that of neurotypical peers in motor planning tasks involving object transport. For example, Fabbri-Destro and colleagues [50] reported that children with ASD did not show significant differences in motor performance in relation to the level of precision requested by the action in a reach to grasp task. A similar result was observed by Forti et al. [51], who found that that unlike neurotypical children, who reduced their hand velocity when nearing the target and started to pre-orient their hand in order to release the object, children with ASD exhibited greater hand velocity when they reached the object. A possible interpretation for these results is that children with ASD may have difficulty using sensory information to guide their actions, which would account for alterations in parameters of reaching in object transport tasks [52].

In conclusion, all improvements in performance in a shape sorter task depend on several crucial developments, e.g., increased motor and perceptual competence. Our results confirm that precision demands strongly influence motor performance in both LR and HR children. Although we did not find any significant differences in performance between LR and HR children, observation of the three HR children later diagnosed with ASD suggests the possibility of early-emerging differences in motor planning skills in these children, differences that merit further consideration in future work with larger samples.
